# The validity of laser diffraction system to reproduce hydrometer results for grain size analysis in geotechnical applications

**DOI:** 10.1371/journal.pone.0245452

**Published:** 2021-01-14

**Authors:** Hamzah M. Beakawi Al-Hashemi, Omar S. Baghabra Al-Amoudi, Zain H. Yamani, Yassir M. Mustafa, Habib-ur-Rehman Ahmed

**Affiliations:** 1 Department of Civil Engineering, University of Bahrain, Isa Town, Kingdom of Bahrain; 2 Department of Civil and Environmental Engineering, King Fahd University of Petroleum and Minerals, Dhahran, Saudi Arabia; 3 Department of Physics and the Center of Excellence in Nanotechnology, King Fahd University of Petroleum and Minerals, Dhahran, Saudi Arabia; China University of Mining and Technology, CHINA

## Abstract

The grain size analysis plays a significant role in any geotechnical study. The grain size analysis, by means of sieving, is usually used for coarse material of particle size > 75 μm. For the fine material; the sedimentation methods are frequently adopted (e.g., hydrometers). Other methods also exist such as electron microscopy, digital image analysis and laser diffraction. The fine geomaterials commonly undergo agglomeration which makes the recognition of individual grain size using digital image analysis or electron microscopy challenging. To facilitate and enhance the grain-size analysis, this study was conducted using the Laser Diffraction System (LDS). Seven samples with different nature (composition and texture) and sources were analyzed by hydrometer and LDS. For LDS, various factors were studied such as air pressure, sonication, dilution, refractive index, and distribution method (volume or number). The results were compared qualitatively and quantitatively based on soil classification systems, fractal dimensions, and other parameters. Furthermore, this study provided a novel criterion to determine which LDS distribution method (volume or number) is to be used depending on the Liquid Limit. A combined sieve-LDS system is recommended to obtain the entire grain size distribution. It is concluded that the LDS is a viable technique that can replace the time-consuming hydrometer method to assess the grain-size distribution.

## 1. Introduction

It is well known that the grain-size distribution (GSD) is a preliminary requirement for any geotechnical investigation. This is ascribed to the fact that the GSD of geomaterials is correlated and interrelated with various physical properties and mechanical behavior. GSD is used in the estimation of hydraulic properties [1], evaluation of the degree of crushing [2], estimation of water retention curves [3], soil classification [4], and determination of desertification potential [5] among others. For coarse materials of particle size > 75 μm, the simple mechanical sieving is usually used to generate the GSD [6]. However, for finer materials, sedimentation-based techniques, such as hydrometer and pipette, are used to complete the GSD curves [7]. Since 1904, Stokes’ law has been used to estimate the sedimentation time for different particle sizes and, hence, both the hydrometer and pipette methods are using Stokes’ law to measure the weight concentration (i.e., in pipette method) and the suspension density (i.e., in hydrometer method) [8].

### 1.1. Hydrometer

In the early 5^th^ century, the oldest description of the hydrometer was introduced by Synesios of Cyrene [9] as a tool to measure the weight of water. In the hydrometer method and based on Archimede’s principle, the particles are assumed to be spherical and their sizes are calculated based on Stokes’ law. The suspended particles, viscous fluid density and the particle’s diameter (i.e., equivalent sphere diameter) affect the sedimentation velocity of the particle and, hence, the particle size can be calculated from Eq (1). Furthermore, the cumulative passing/finer percentages required to generate the GSD are calculated from Eqs (2)–(4).

$$\text{Dia}.=\;\sqrt{\frac{30\times \eta }{980\times (G_{S}-G_{SR})}\times \frac{L_{S}}{T_{i}}}$$ (1)

$$\text{Cumulative}\;\text{Passing}\;(\text{\%})=\;\frac{a_{G}\times R_{C}\times P_{200}}{M_{S}}\times 100$$ (2)

$$a_{G}=\;\frac{1.65\times G_{S}}{2.65\times (G_{S}-G_{SR})}$$ (3)

$${\text{R}}_{C}={\text{R}}_{A}-{\text{C}}_{ZR}+{\text{C}}_{T}$$ (4)

In these equations, Dia. = equivalent sphere diameter of the particle [mm]; ƞ = dynamic viscosity of the suspended medium, which is a function of temperature and its values are tabulated in the standard [7] [poise = g.cm^-1^.s^-1^]; *a*_*G*_ = specific gravity correction factor; G_S_ = specific gravity of the particles; G_SR_ = reference specific gravity for water, taken as 1.0 g/cm^3^ at 4°C; L_S_ = the distance from the suspension surface to the point at which the density of the suspension is being measured, which is a function of the actual reading plus the average meniscus reading due to surface tension (taken as 1.0 g/l in this study), and tabulated in the ASTM standard [cm]; T_i_ = the cumulative time interval from the beginning of the sedimentation (minutes); P_200_ = cumulative passing ASTM sieve #200 (~ 75 μm) of the particles (taken as 1.0 for this study); M_S_ = dry mass of the particles used (i.e., 50 g in this study); R_A_ = actual hydrometer (i.e., type 152H) readings [g/l]; C_ZR_ = reference zero reading correction measured before the particles are added [g/l]; C_T_ = reading correction for temperature, tabulated in the ASTM standard [g/l].

Despite the fact that the hydrometer technique is standardized, many deem it to be, technically and practically, demanding and time-consuming. Such a claim is manifested when dealing with samples having a size smaller than 2 μm (i.e., the size of the clayey materials and colloids) [10], where the readings may continue for 96 hours in some cases [7] due to the Brownian motion effects. Moreover, sample preparation, the addition of a dispersing agent, and the interpretation of the result require a well-trained technician. Also, there are other disadvantages including the sensitivity of the test to any vibration, which would affect the sedimentation rate. Further, the hydrometer method is limited to a range of constant specific gravity (G_S_) of 2.45 to 2.85, a particle size within 1 to 75 μm, and any particle of smaller size is not usually detected by this method [10]. Also, it is reported in the literature that the sedimentation methods slightly overestimate the fine material content in the samples (i.e., the bias in clay fraction) [11]. The influence of such drawbacks becomes of significance in projects where time and rapidity of measurements are critical factors.

### 1.2. LDS

The laser diffraction system (LDS) is another method that is used to analyze the particle size in suspension or dry forms at a particle size ranging from nanometers to a few millimeters [12]. The concept behind LDS follows Mie’s scattering theory (recommended for small/fine particles) or any other approximation methods for Mie’s theory (such as Rayleigh or Fraunhofer approximations), where the wavelength of the laser (or light) is comparable to the scattered particles [13]. Mie’s theory assumes a volume-equivalent sphere model in the GSD calculations. However, other shape models can be solved through recently developed algorithms. The GSD is measured through the LDS by measuring the scattered intensity of the laser beam that passes through a dispersed sample [14].

In LDS, as in Rayleigh approximation, for example, the particle size/diameter (d) is determined based on the initial and scattered intensity of the laser beam (I), the distance between the particle and detector (R), scattered angle (θ), the wavelength of the laser (λ), and refractive index of the particles (n), as shown in Eq (5) [15]. The particle size and distribution can be calculated on volume-basis (sensitive to large particles), as shown in Eq (6), or on number-basis (sensitive to small particles), as shown in Eq (7). A correction for the passing percentages can be applied for LDS in a way similar to the hydrometer method shown in Eq (2), as will be discussed later.

$$\text{I}={\text{I}}_{0}(\frac{1+{\text{cos}}^{2}\theta }{2R^{2}})(\frac{2\pi }{\lambda })(\frac{n^{2}-1}{n^{2}+2})\;{\left(\frac{Dia.}{2}\right)}^{6}$$ (5)

$$\text{VMD}=\;\frac{\sum (Dia.^{4}\times n_{i})}{\sum (Dia.^{3}\times n_{i})}$$ (6)

$$\text{NMD}=\;\frac{\sum (Dia.\times n_{i})}{\sum (n_{i})}$$ (7)

Here, Dia. = Equivalent sphere diameter of the particle (mm); I = Scattered laser beam intensity (cd); I_0_ = Initial laser beam intensity (cd); θ = Scattered angle (degrees); R = Distance between the particle and detector (mm); λ = Wavelength of the laser (mm); n = Refractive index of the particles; VMD = volume mean diameter (mm); n_i_ = number of particles; NMD = number mean diameter (mm).

It is to be reported that the LDS can detect particle sizes in the range of approximately 0.02 μm to 2 mm, based on the instrument type and setup (Microtrac FLEX Software User Manual, USA). It is to be noted that the light scattering technique, in general, was discussed in some standards [14, 16], but not for geomaterials. For several years, research has been conducted to improve the knowledge of light scattering methods for particulate systems. Therefore, the technique is quite known for GSD analysis. However, further research is required to validate the applicability of LDS to geomaterials. This paper, thus, aims at verifying the reliability of the LDS technique as a replacement for the hydrometer method, based on the results of an experimental work carried out on 7 different samples of geomaterials that differ in nature and collected from different sources.

## 2. Experimental procedure

### 2.1. Materials

Seven different samples (e.g., clay minerals, cement kiln dust, limestone powder, and marl soil) collected from different locations were used in this study, as summarized in Table 1. As shown in Table 1, all **S1**, **S6**, and **S7** are characterized as marl, which is the predominant type of soil in eastern Saudi Arabia and is being used in most of the construction projects. Therefore, different samples were collected from different sources covering different ranges of plasticity. Moreover, two different types of Bentonite (i.e., **S2** and **S5**), that are being locally utilized, were also used in this study. Lastly, two of the most common types of stabilizers (i.e., cement kiln dust (**S3**) and limestone powder (**S4**)) were included to increase the variety of the geomaterials used in the current study. Therefore, and for simplification, the materials will be categorized into three main groups: marl, stabilizers and bentonite.

**Table 1:** Samples information basic properties.

Sample ID	Sample Description	Location	G_S_	LL (%)	PL (%)	PI (%)	Plasticity [29]
**S1**	Marl	Saudi Arabia, Eastern Province	2.461	68.7	21.3	47.4	Very High
**S2**	Commercial Bentonite/Montmorillonite Clay	Saudi Arabia, Western Province	2.338	434.4	52.9	381.5	Very High
**S3**	Cement Kiln Dust (CKD)		2.688	55.4	19.4	36.0	High
**S4**	Limestone Powder	Saudi Arabia, Eastern Province	2.455	29.6	19.3	10.3	Medium
**S5**	Commercial Bentonite/Montmorillonite Clay	India	2.489	260.3	44.5	215.8	Very High
**S6**	Marl	Saudi Arabia, Eastern Province	2.281	35.7	18.3	17.4	Medium
**S7**			2.304	28.8	15.4	13.4	Medium

All the samples were air-dried at 30 °C for 24 hours and then oven-dried at 110 ± 5 °C for another 24 hours to ensure moisture removal. Thereafter, the samples were sieved on ASTM sieve #200 (~ 75 μm) in preparation for both hydrometer and LDS tests.

### 2.2. Testing methods

#### Specific gravity

The specific gravities (G_S_) of the samples were determined in accordance with ASTM D854 [17], as shown in Table 1. The determination of (G_S_) values for such fine materials is a rigorous and time-consuming procedure that involves boiling, blending, and applying vacuum simultaneously for a minimum duration of 3 hours to expel the air bubbles from the samples. The G_S_ was determined to be used in particle size calculations for the hydrometer method.

#### Atterberg limits

In order to classify the samples in terms of plasticity, liquid limit (LL), plastic limit (PL), and plasticity index (PI) were obtained in accordance with ASTM D4318 [18]. The Atterberg limits are listed in Table 1.

#### Hydrometer

The hydrometer (i.e., type 152H) method, as per the ASTM standard, was used, as discussed earlier. A 50 g sample from each material is placed in a 250-ml beaker filled with a 125-ml of the dispersing agent solution (Sodium Hexametaphosphate) with a 40 g/liter concentration. The mixture is then stirred thoroughly (10,000 RPM) until the material is properly wetted. Then, the mixture is allowed to soak for about 16 hrs. Thereafter, the sample is stirred again for a minute, and then it is placed in the sedimentation cylinder. Then, the cylinder containing the sample is filled with distilled water until the total volume is 1000-ml. For proper agitation of the slurry; the cylinder is closed with a rubber stopper in the open-end, and the cylinder is turned upside down and back for a minute. Finally, the hydrometer readings are continually taken for longer than a full day. Lastly, the GSD is obtained using Eq (4).

### 2.3 Laser diffraction analysis

For the current study, the Microtrac S3500 LDS instrument was used, which has a Tri-laser system of 780 nm wavelength and able to detect particle sizes in the range of 0.02 μm to 2.8 mm. The time run of each analysis was 45 seconds. Samples were added to the device through the sample cell where the laser is transmitted through the Fourier lens. However, laser diffraction analysis is useful only if the samples are prepared in a manner that all the particles are separated and de-agglomerated. With that in mind, there are two main approaches for sample analysis in LDS, those are the wet approach, where the samples are diluted using specific solvents, and the dry approach where the samples are carried through air steam [19]. In the dry approach, the soil samples can be dispersed by increasing the air pressure which mainly depends on the device design [20]. With the latest development in laser diffraction devices, it is possible to detect particle sizes, using the dry approach, in the range 0.1 to 3500 μm [20]. As for the wet approach, it was reported that it is recommended to use such an approach for cohesive powders since it will help in de-agglomerate and disperse the soil particles [21]. The samples were replicated 3 times and the differences in the mean size among the replicates were found to be < 5% for all the samples. The third replicates are recorded and presented in the study.

The laser scattering is based on the classical Mie’s theory (spherical particles; comparable with the Hydrometer assumptions), which is directly dependent upon the optical properties of the materials such as the refractive index [13]. The real refractive index (n_r_) is defined as the ratio of the light speed in vacuum to the phase velocity of the light in a medium, and it is usually measured at a wavelength of 589 nm at 20°C. However, the measurements of the n_r_ for powders are challenging and might be conducted through immersion or optical diffraction methods [22, 23]. Moreover, for geomaterials, the determination of the refractive index is more difficult due to the size, optical, compositional, and geometrical anisotropy as well as the heterogeneity of geomaterials. However, remote sensing technologies reveal an apparent real refractive index for soils in a range of 1.42 to 1.73 [24].

In the literature, the maximum difference between the measurements of GSD of geomaterials using laser scattering was reported as 1.9% using different values of the real refractive index, while the imaginary refractive index effects were found to be negligible [11, 25]. Further, one Bentonite sample (i.e., **S2**) was selected to investigate the effect of the real refractive index on LDS. This is because this sample has the least possible impurities (Bentonite mineral is the dominant composition), as stated by the distributor. Then, two identical samples have been analyzed in the LDS instrument under two different values of the refractive index (n_r_) to investigate (n_r_) effects on the measurements.

#### Verification of reproducibility

Regarding the verification of the reproducibility of the LDS instrument, a standard/reference material provided by the manufacturer (Microtrac, Inc., USA) was tested in a water solution with a concentration of 1.4 g/l (which is the concentration selected in this study for the analysis as will be discussed later) by the LDS instrument (Microtrac S3500). The standard material is the transparent glass beads powder (ID: 159666) with certified properties such as 1.51 refractive index, 2.5 specific gravity, and mean size range of 56.0 to 60.1 μm.

#### Samples preparation and pretreatment

In order to carry on the laser diffraction analyses, several pretreatments and trials were carried out to select both the proper sample size and dispersion approach. Toward that end, both samples dispersion and quantities (i.e., concentration) were analyzed as follow:

- Samples Dispersion: As mentioned earlier, there are two approaches at which the particle dispersion could be achieved, those are wet and dry. Therefore, for the current study, the effect of both approaches was assessed using the **S1** sample. The sample was first dispersed for the dry analysis using two different pressures within the limit of the device (i.e., 1 and 3 bars). Then, for the wet analysis, the sample was dispersed using either the dispersing agents as in the hydrometer analysis, distilled water, or the ultrasonic energy applied to a diluted solution of the sample.
  The sound energy was applied to the sample in distilled water solution using an ultrasonic probe with a frequency about 20 kHz for 120 seconds. However, for geomaterials, the dispersing process may be altered by sonication and causing adverse effects [26]. It was reported that the non-clayey materials are the most susceptible to particle damage/breakage [26], therefore, the effect of sonication on the dispersion of particles was studied for **S1** sample in a distilled water solution with 50 g/l concentration in a way similar to a hydrometer sample. Also, with the same concentration, the **S1** sample was studied for the effect of the addition of a dispersing agent (40 g/liter Sodium Hexametaphosphate, as used in the Hydrometer case) to the solution.
- Sample Concentration: The effect of the dilution was also studied for the **S1** sample with concentrations of 50, 7.0, 1.4, and 0.28 g/l. The dilution of the samples is meant to keep the sample quantity that is required for testing the least possible which can be advantageous for the limited sources samples (such as lunar or extraterrestrial soils), among other advantages. The samples were diluted before being fed into the sample delivery controller (SDC) attached to the LDS instrument. The feeding of the samples was done using 2 ml disposable pipet droppers. All the concentrations were within the safe loading index range (0.1 to 100), as reported in the LDS instrument manual (Microtrac, Inc., USA). Based on the findings of the sample concentration study conducted on **S1**; the remaining samples (**S2 to S7**) were analyzed in the LDS accordingly. Mathematical manipulation of the LDS results was also undertaken to study the effects of volume and number distributions.

## 3. Results and discussion

### 3.1. Grain size distribution using hydrometer

**Fig 1:**
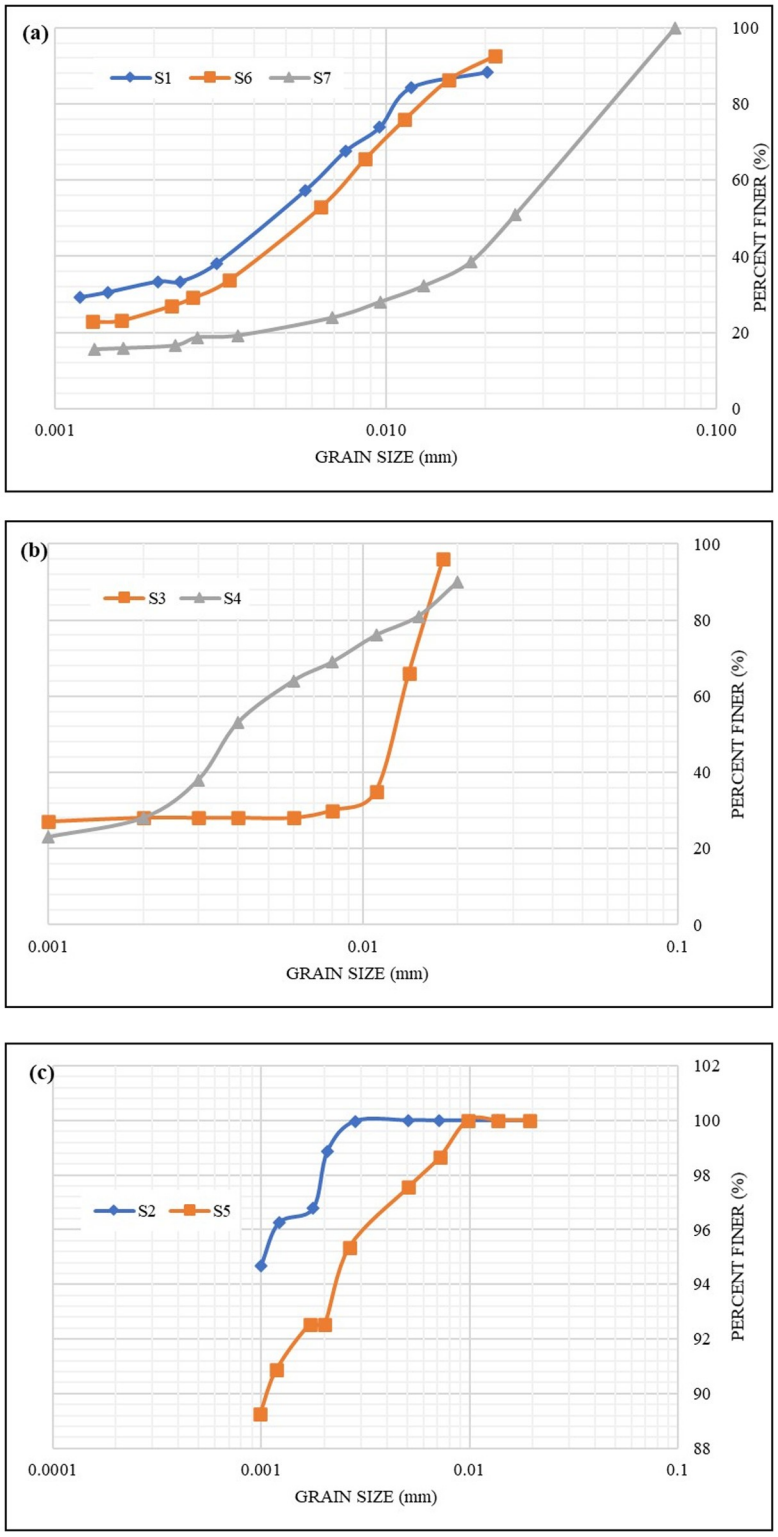
Grain size distribution of a) Marl soils; b) Stabilizers; and c) Bentonite.

Fig 1 shows graphically the grain size distribution (GSD) for the seven samples using the results of the hydrometer tests. The samples were divided into three main groups for better comparison, namely: marl soils (i.e., **S1**, **S6**, and **S7**), stabilizers (i.e., **S3** and **S4**) and Bentonite (i.e., **S2** and **S5**). Fig 1a shows the GSD of marl soils whereby the majority of particles’ sizes are smaller than ~ 20 μm for both **S1** and **S6**. For **S7**, particles’ sizes are smaller than 75 μm. As for both the bentonite and the stabilizers, Fig 1b and 1c show that all particles’ sizes are smaller than ~ 20 μm. However, the smallest calculated size for all the samples was 1 μm. Such results confirm the hypothesis that a hydrometer analysis can only detect particles larger than 1 μm, as mentioned in Section 1.1. Therefore, it is essential to apply the laser diffraction analysis to measure smaller sizes.

### 3.2. Grain size distribution using LDS

#### LDS calibration

As mentioned in Section 2.3, and to apply the laser diffraction analysis, the LDS device was calibrated using the referenced material (glass beads powder) in a distilled water solution with a concentration of 1.4 g/l, where the wet LDS was used. The glass beads have been used because they have more uniformity and homogeneity than the geomaterials, which makes the reproducibility evaluation clearer and isolated from other influential factors. It should be noted that for the current study, the concentration of 1.4 g/l was selected based on many trials that were carried out on sample **S1**, as will be discussed later. Therefore, the same concentration was used for the LDS calibration to ensure that all the samples would be tested in the same conditions. The carrier medium for this analysis was distilled water of n_r_ = 1.33. The analysis yielded a mean size of 57.1 μm, which falls within the stated range by the manufacturer. Therefore, the reproducibility and controlled calibration of LDS apparatus has been verified.

#### Refractive index

**Fig 2:**
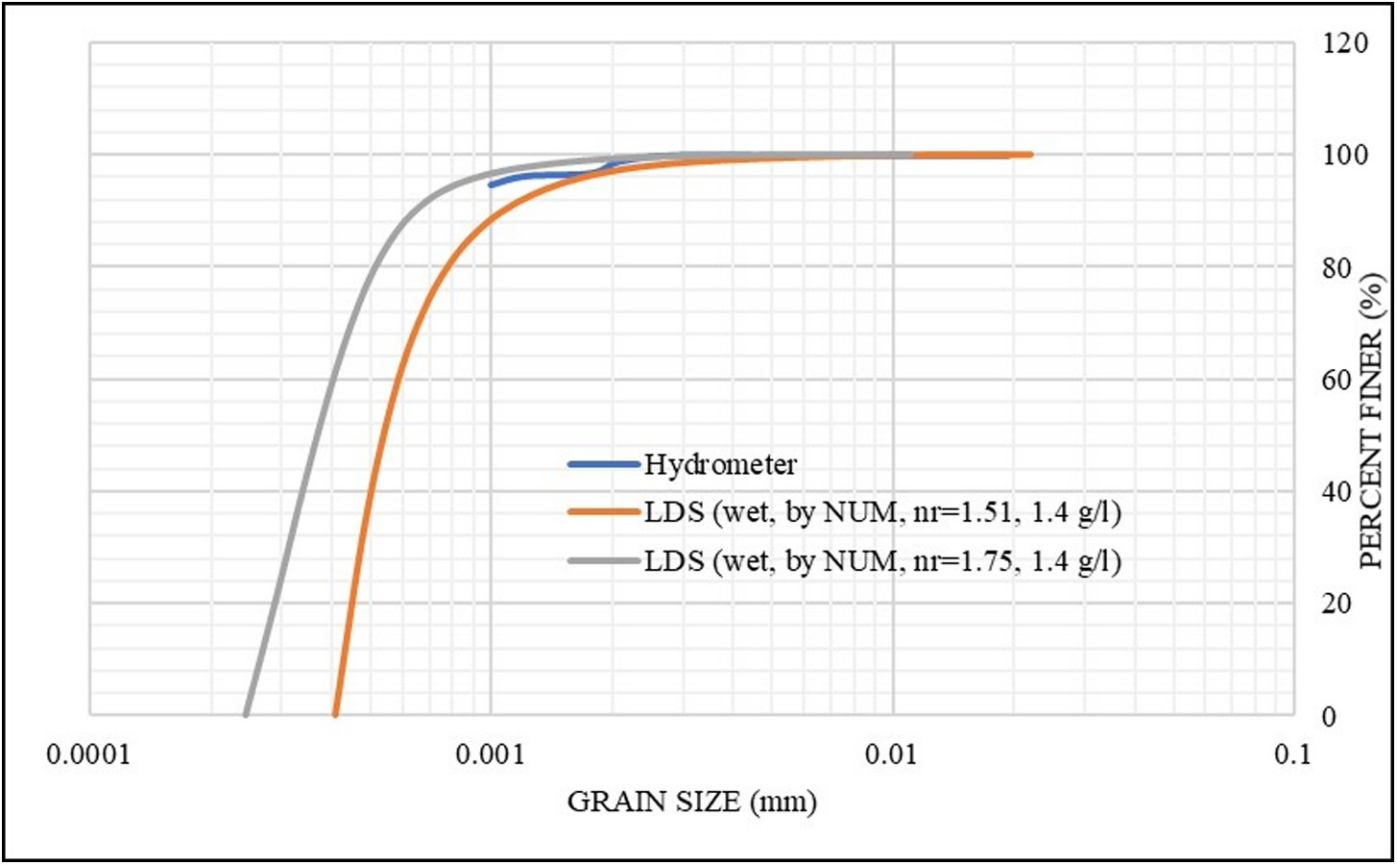
Effect of refractive index on the grain size distribution for S2.

**S2** sample (refer to Table 1) was selected to study the effect of changing the refractive index (n_r_) on the grain size analysis, as discussed in Section 2.3. The refractive index (n_r_) was arbitrarily changed in the LDS instrument setup to investigate its effects to compare it with the hydrometer results. Two refractive index values [i.e., 1.75 and 1.51 that fall within the reported range of soils] were explored for the diluted samples in a distilled water solution with a concentration of 1.4 g/l to yield the best matching results with the hydrometer analysis. The results are shown in Fig 2, and the mean standard error (SE), between the measurements (i.e., passing% for each particle size) using the two different refractive index values, was insignificant (0.37%). Therefore, the (n_r_) value of 1.75 was maintained for all the samples in this investigation.

#### Dry analysis

**Fig 3:**
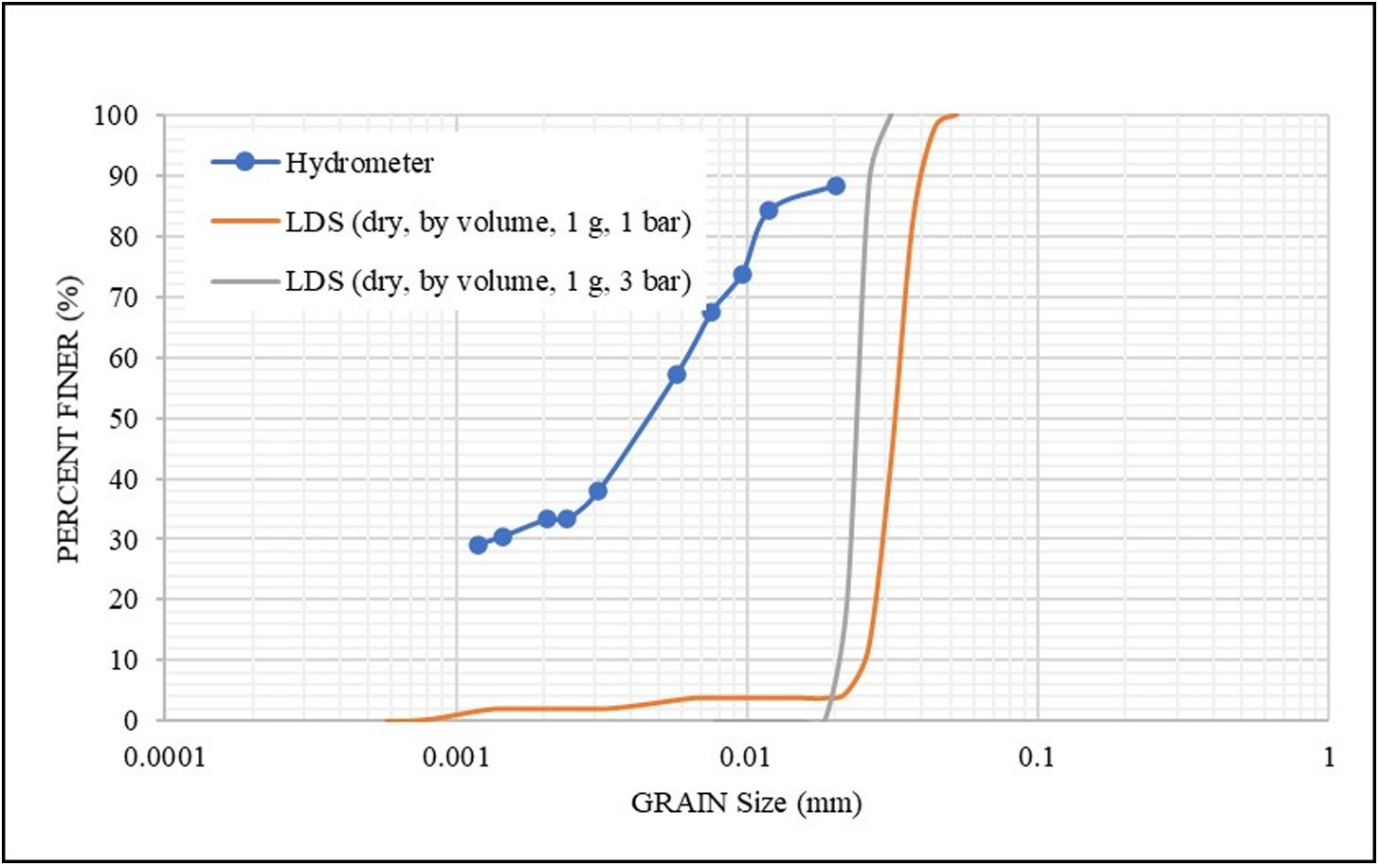
Effect of vacuum pressure on the grain size distribution for S1.

As mentioned in Section 2.3, the LDS instrument provides a dry measurement option for the materials. Therefore, two **S1** samples (1 g each) were analyzed in LDS using two different pressures (1 and 3 bar), as shown in Fig 3. It is shown that the use of higher pressure (3 bar) reduced the size of the particles (due to dispersion) towards the hydrometer results, but such a pressure is expected not to overcome the strong bonding between the particles. Therefore, the dry analysis method was not applied to the other samples.

### 3.3. Samples dispersion and concentration (wet analysis)

#### Sonication

**Fig 4:**
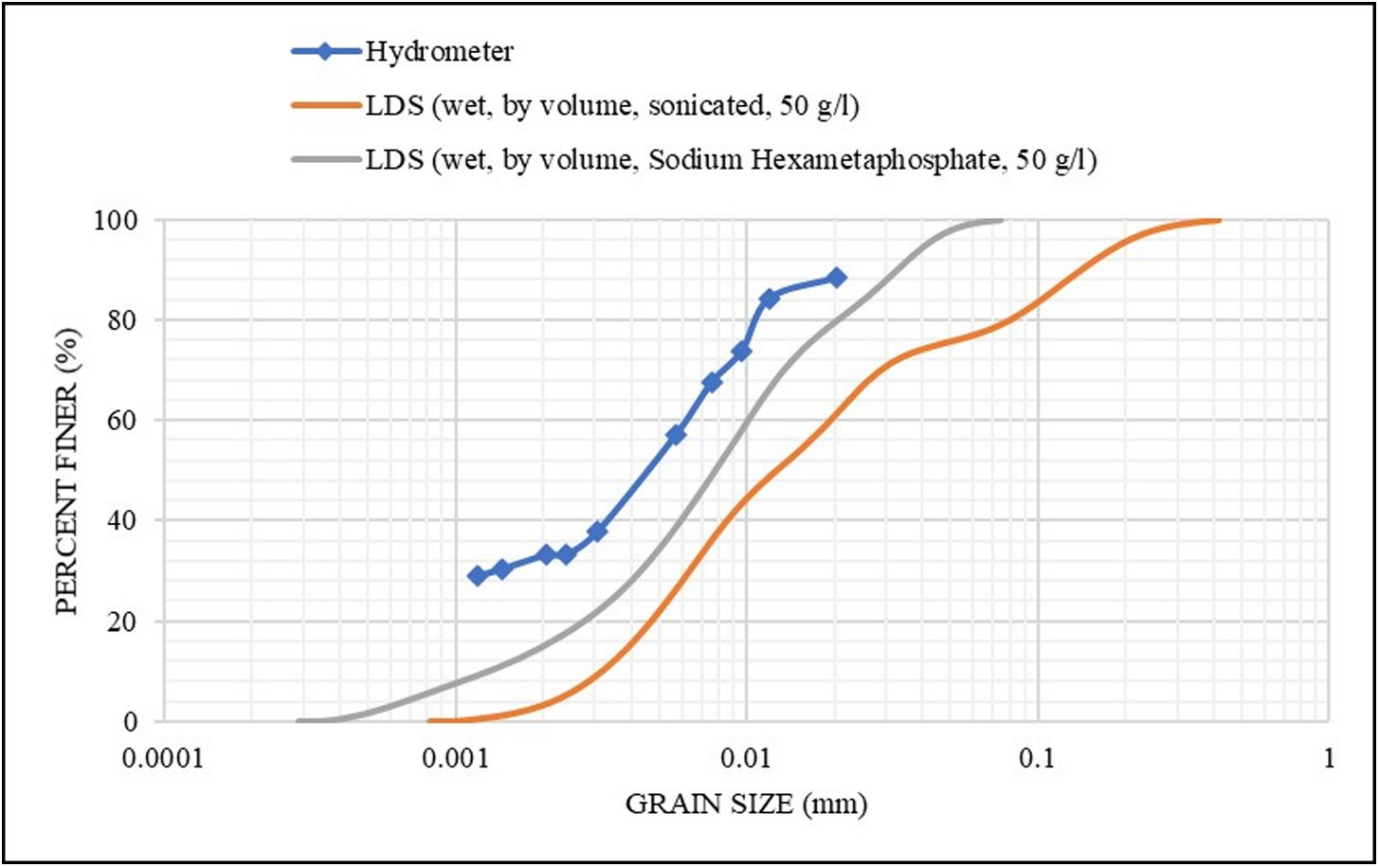
Effect of sample dispersion by sonication & Sodium Hexametaphosphate on the grain size distribution for S1.

In order to achieve the most reliable dispersing approach, sample **S1** was dispersed by both sonication and sodium hexametaphosphate separately. The sonicator probe was applied for 300 seconds [26] for dispersing the particles, as an alternative method to using a dispersing chemical agent. However, and as shown in Fig 4, the sonication produced larger size results (i.e., adverse effects). This effect could be ascribed to physical damages (e.g., alteration of surface charges) of the particles from the induced energy, which might have agglomerated the damaged particles when the sonication was stopped [26]. Therefore, the sonication was not applied to the other samples.

#### Dispersing agent

Using a concentration of 50 g/l of **S1** sample (similar to the hydrometer) and sodium hexametaphosphate as a dispersing agent, the LDS measurements are presented in Fig 4. The results were found to be close to the hydrometer results. However, the dispersing agent was not added to the other samples for the reasons shown later (since this study was aimed to provide accurate results with the least possible efforts and sample amount).

#### Dilution

**Fig 5:**
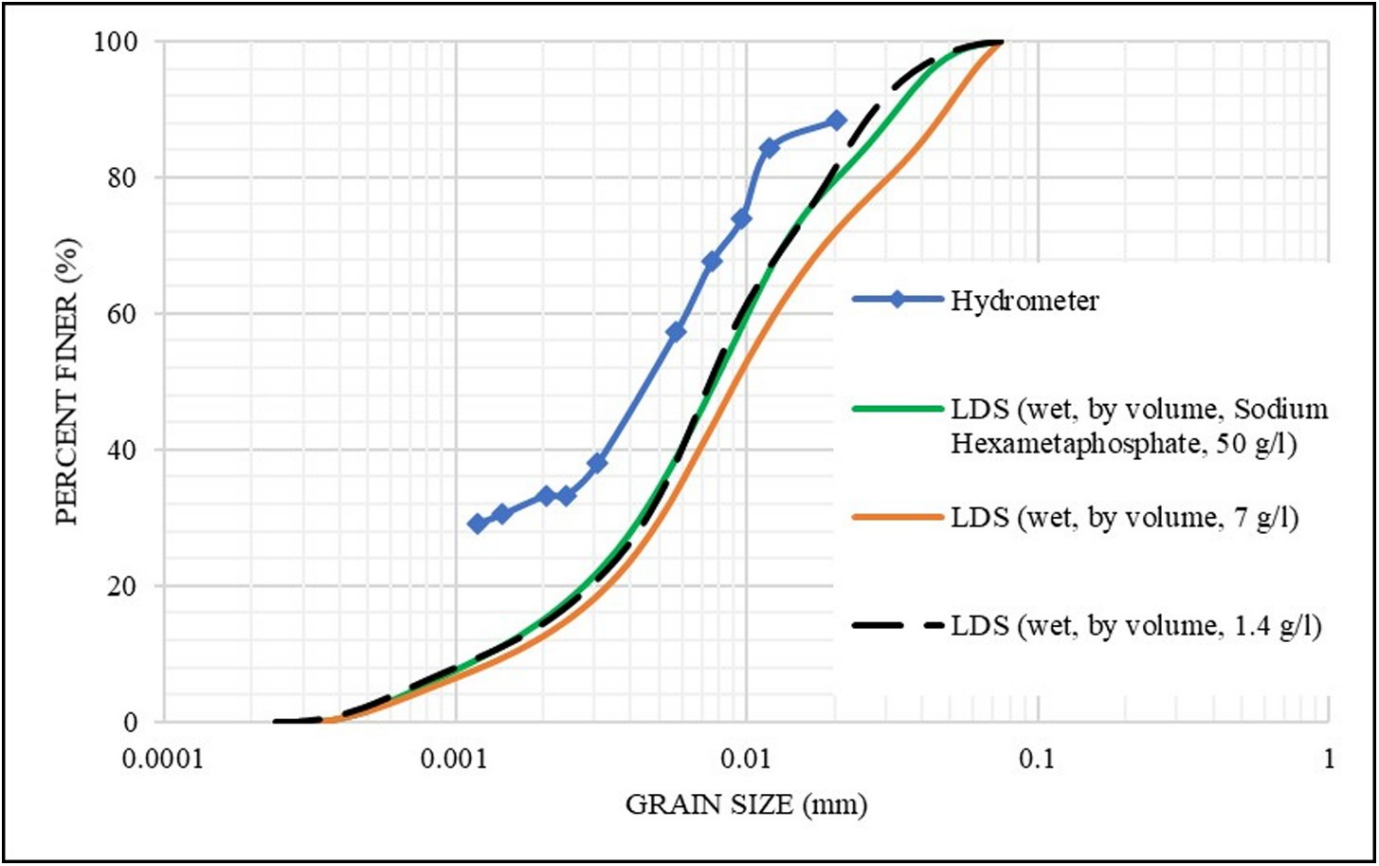
Effect of sample concentration on the grain size distribution for S1.

Different concentrations (7, 1.4, and 0.28 g/l) of the **S1** sample in a distilled water solution were tried, to select the least possible concentration of the sample detected by the instrument to overcome the multiple scattering and agglomeration issues. The concentration of 0.28 g/l was not detected by the instrument and, therefore, was not applied to the other samples. As shown in Fig 5, the dilution improved the overall LDS results (i.e., more matching with hydrometer results). The concentration of 1.4 g/l yielded the closest results to the hydrometer results and was almost matched with the dispersing agent results for the 50 g/l concentration. Therefore, and for the sake of simplifying the testing and sample preparation; all the other samples were analyzed in a distilled water solution (wet LDS) at a concentration of 1.4 g/l.

### 3.4. Data analysis

#### Number and volume distributions

**Fig 6:**
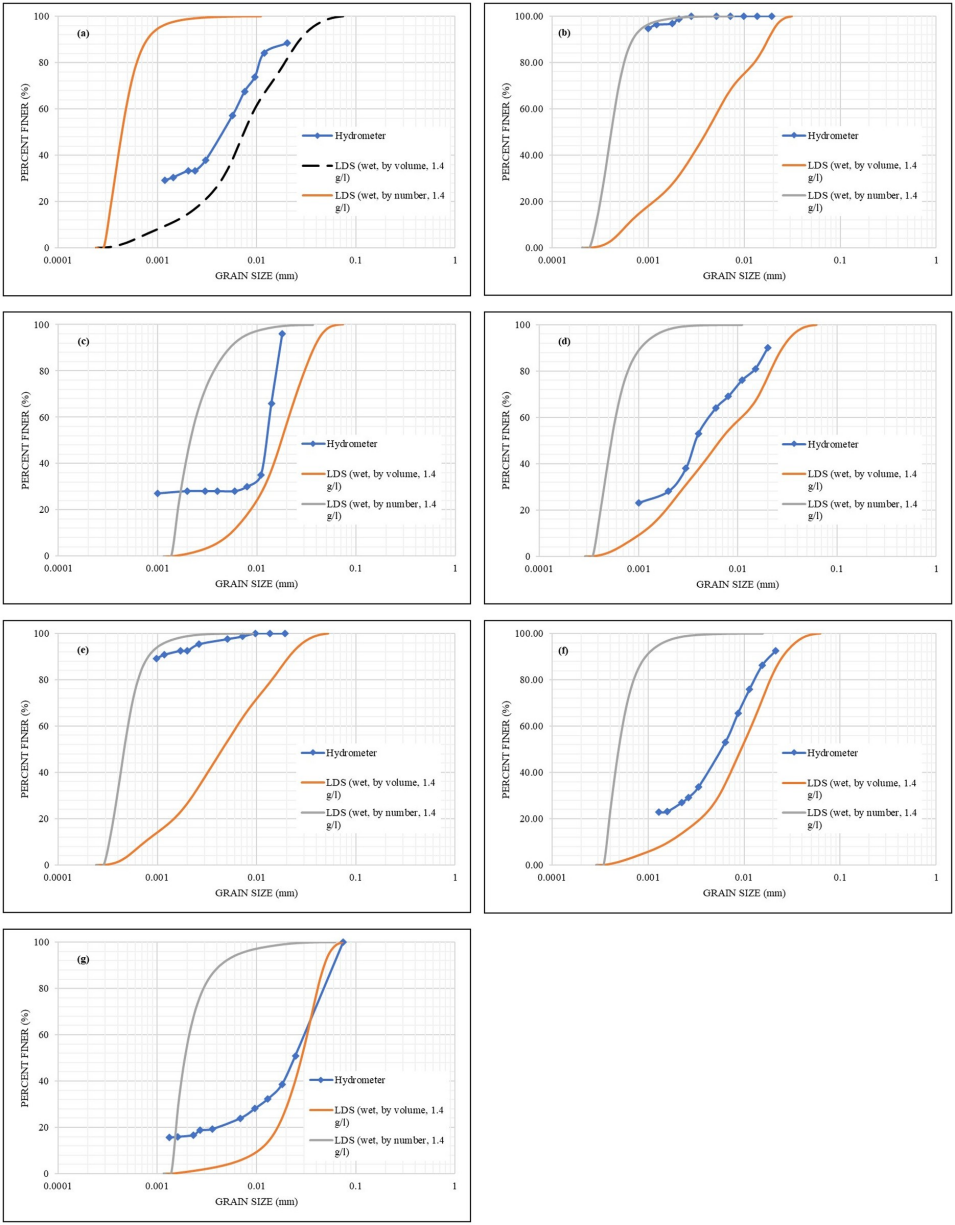
Comparison of the volume, number and hydrometer distribution for a) S1 b) S2; c) S3; d) S4; e) S5; f) S6; g) S7.

**Fig 7:**
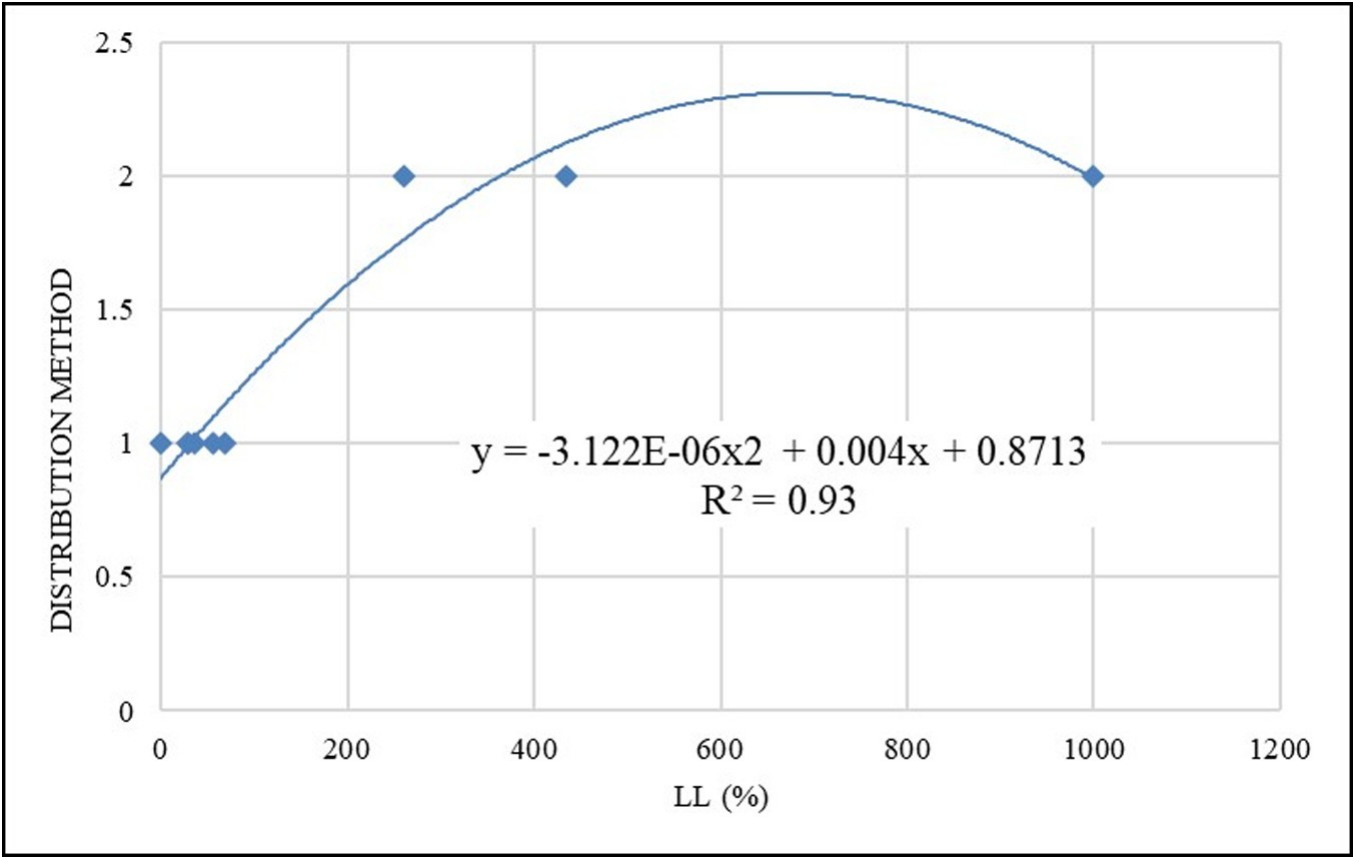
Distribution-method predictive model.

**Fig 8:**
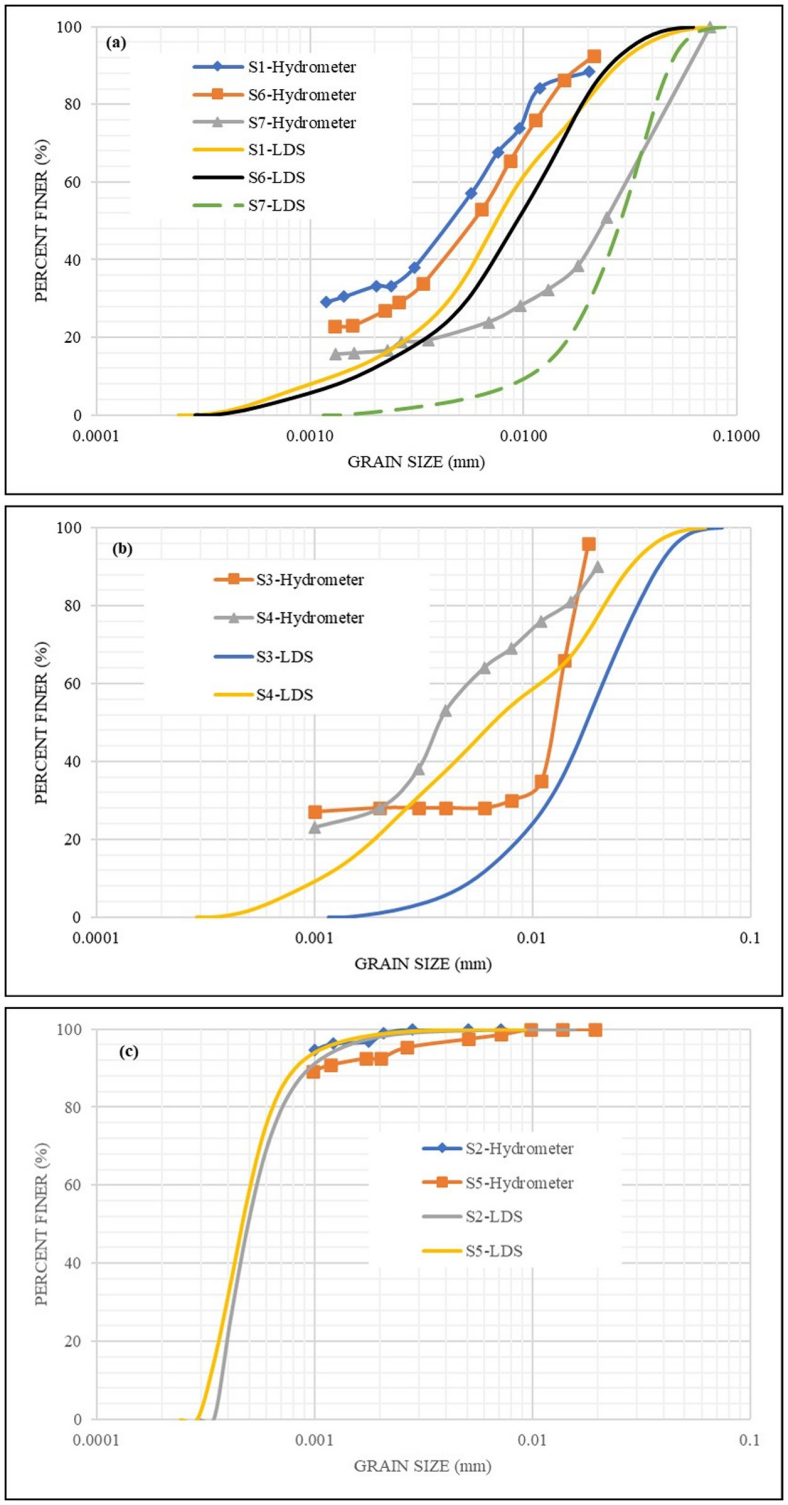
Grain size distribution of a) Marl soils; b) Stabilizers; and c) Bentonite, for both hydrometer and LDS.

The selection of the distribution method is based on the sensitivity required either for small particles (number) or large particles (volume), as addressed in Eqs (6) and (7). For the **S1** sample analyzed by wet LDS with a concentration of 1.4 g/l in a distilled water solution, the volume and number distribution results are presented in Fig 6a. It is worth mentioning that the number distribution results of a sample are usually smaller than that of the volume distribution results of the same sample, refer to Eqs (6) and (7). The use of those methods can overcome the light scattering sensitivity issues of small particles that are reported in the literature [27]. However, the question arises as to when to use which? To answer this question, all the other samples were analogously analyzed, and both the number and volume results were obtained as depicted by Fig 6b–6g. It was found that only in the case of **S2** and **S5** samples (Fig 6b and 6e), the number of results did yield closer matching with that of the hydrometer. Therefore, a stepwise statistical (regression) analysis was conducted to correlate the method of distribution (either number or volume) with the properties of the sample (G_S_, LL, PL, and PI). The liquid limit (LL) was found to be highly correlated with the method of distribution that is the closest to the Hydrometer results (by assigning a value of 2 for the number method, and 1 for the volume method), and a quadratic model is provided in Eq (8) and Fig 7. Therefore, the method of the distribution of any geomaterial can be determined/selected based on its liquid limit. The final and best correlations of the LDS results with the hydrometer results of all the samples are shown in Fig 8. Based on the provided equation; any sample with LL greater than or equal to 180% will result in the number method. However, as a recommendation; this intermediate (breakthrough) range is to be further verified by future studies. Moreover, further studies are required to evaluate the effects of the mixed origin or size samples on the method of distribution. Nevertheless, the provided equation is valid for the liquid limit range used in the current study.

$$\text{Method}=\;0.871+[0.004\times \text{LL}]-[LL^{2}\times (3.122E-06)],\;\text{R-Squared}=0.93$$ (8)

Where: Method = distribution method, rounded to the nearest whole number (either 1 for volume method, or 2 for number method); LL = liquid limit (%, valid for a range of 0 to 1000; assuming that any geomaterial sample with a LL > 434.4% will follow this model).

#### Representative sampling

It was reported in the literature that it is always challenging to obtain a representative sample for light scattering measurements in general [28]. Such a challenge might be the main reason that this technique cannot replace the sieve-hydrometer system as a whole. However, the authors propose the use of a sieve-LDS system instead. Regardless of the flaws of the sieving technique, it is standardized, simple, fast, and considered acceptable to obtain the GSD for coarse materials (> 75 μm). For a given sample, the sieving is first recommended. Then, the material passing the ASTM sieve #200 (~ 75 μm) is collected for LDS analysis and, hence, the heterogeneity of the material will be reasonably minimized, and the sample is expected to be more representative. Thereafter, the LDS cumulative passing percentages (only for 75 μm sizes and smaller) can be corrected to be connected with the GSD obtained by the sieve and, hence, the complete GSD information is obtained. The suggested correction is shown in Eq (9). In this study, only the LDS result of the **S1** sample shown in Fig 1c has slightly to be corrected.

$${\text{Corrected}\;\text{Cumulative}\;\text{Passing}}_{LDS}(\text{\%})=(\frac{P_{200}}{P_{200LDS}})\times [Actual\;Cumulative\;Passing_{LDS}\;(\%)]$$ (9)

P_200_ = cumulative passing ASTM sieve #200 (~ 75 μm) of the particles (%); P_200LDS_ = equivalent cumulative passing LDS size (~75 μm) of the particles (%).

#### Fractal dimension

**Fig 9:**
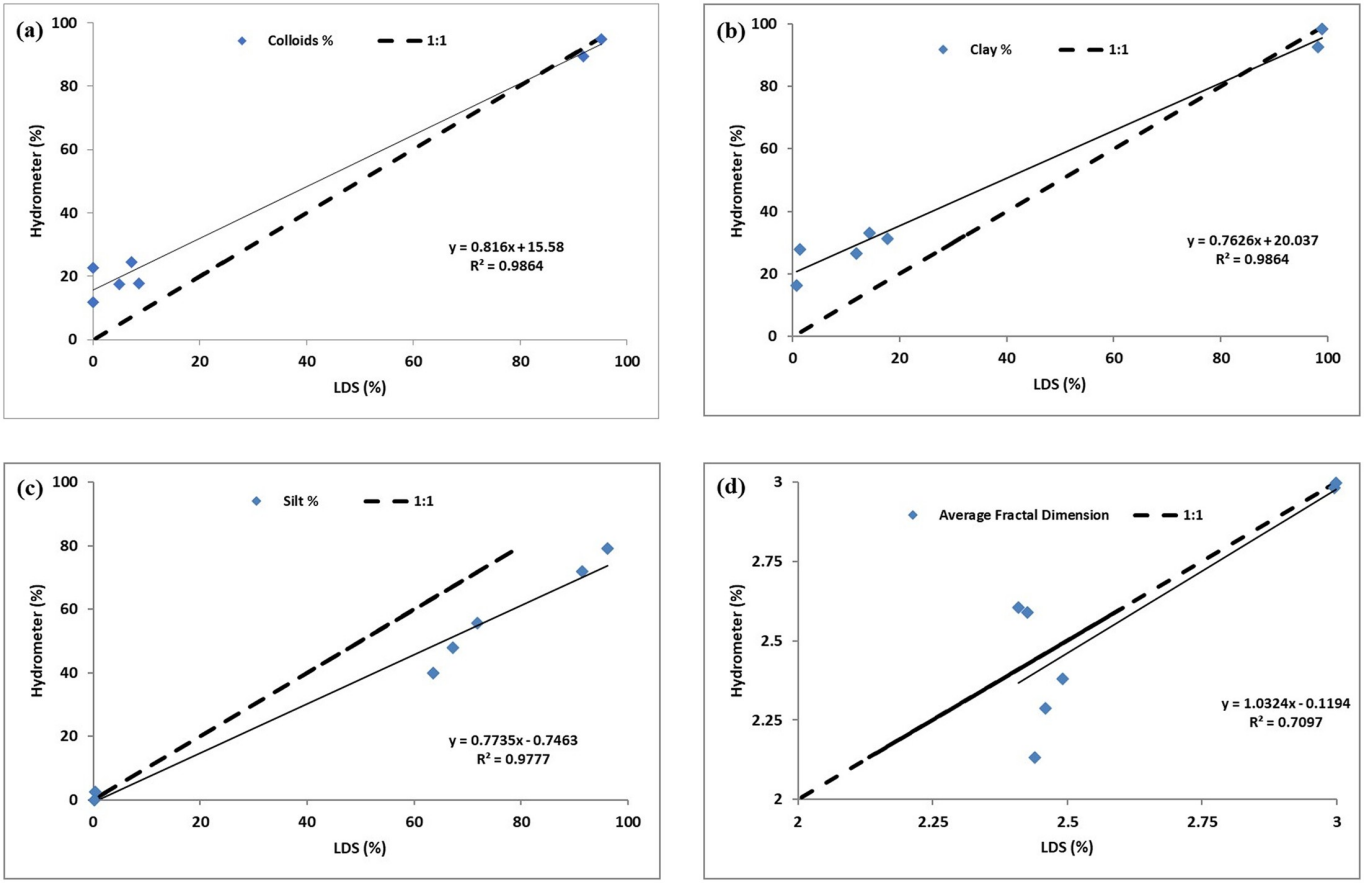
Comparison between LDS and hydrometer methods based on (a) Colloids %, (b) Clay %, (c) Silt %, and (d) Fractal dimension.

**Fig 10:**
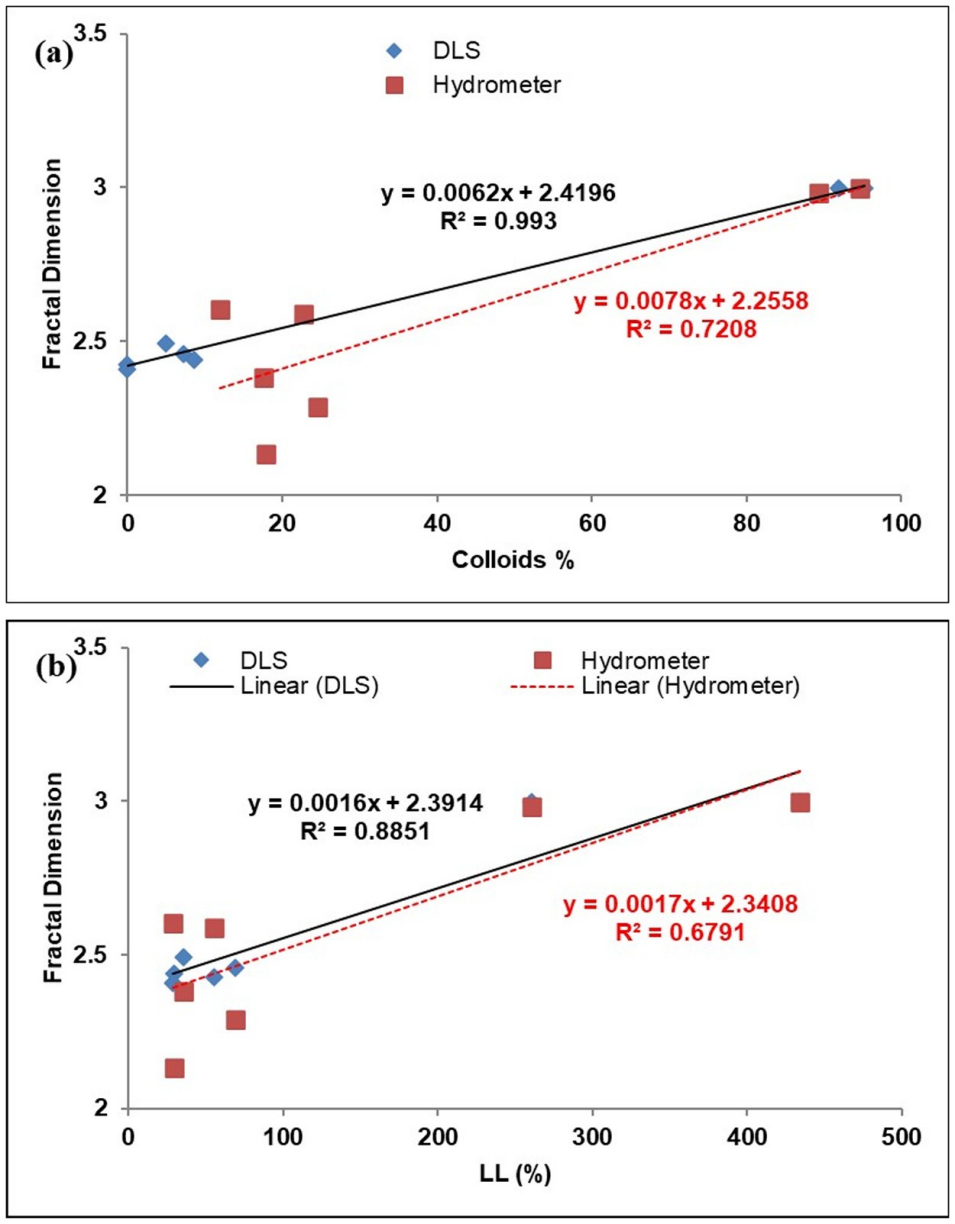
Fractal dimension correlations with (a) Colloids % & (b) LL %.

The fractal dimension is a single-valued dimensionless quantity that can be used for irregular geometries [29]. It can represent the GSD in geomaterials, especially the soils, and it usually ranges from 2.0 to 3.0, as reported in the literature [5]. It is found to increase as the fine particles in a sample increase. The fractal dimension of the GSD obtained by the hydrometer and LDS for all of the samples was calculated from Eq (10) [5], and the average fractal dimension for each sample is summarized in Table 2. The fractal dimension values calculated from LDS and hydrometer results are in good agreement with a mean standard error (SE) of 0.07, as shown in Fig 9d. Both, the LDS and hydrometer results show a similar trend with the fractal dimension. However, the LDS results were found to be more consistent with the fractal dimension than the hydrometer, which can be ascribed to the fact that the experimental error in the LDS is expected to be significantly less than that in the Hydrometer case. The fractal dimension values calculated from LDS results are highly correlated (based on R-squared values) than those from the hydrometer results for the percentage of colloids (defined as particles with a diameter of less than 1 μm), as shown in Fig 10a, and with the liquid limit, as shown in Fig 10b.

$$0\;<\text{Fractal}\;\text{Dimension}=\frac{\left[3\times {\text{log}}_{10}(\frac{d_{m}}{d_{max}})]-{\text{log}}_{10}(\frac{Material\;Percentage\;(\%)}{100}\right)}{{\text{log}}_{10}(\frac{d_{m}}{d_{max}})}\;\leq 3.0$$ (10)

Where: d_m_ = the average material size (for sand: 2.41 mm, silt: 0.04 mm, clay: 0.001 mm) [4]; Material Percentage = the percentage of (sand, silt, or clay) in a sample; d_max_ = the maximum particle size in a sample (taken as 0.075 mm for this study).

**Table 2:** Comparison between LDS and hydrometer based on soil classification and fractal dimension.

Sample ID	Soil Classification [4]				Fractal Dimension	
	USCS		AASHTO			
	LDS	Hydrometer	LDS	Hydrometer	LDS	Hydrometer
**S1**	CH^a^	CH	A-7-6 (54) ^c^	A-7-6 (54)	2.46	2.29
**S2**	CH	CH	A-7-5 (457)	A-7-5 (457)	3.00	3.00
**S3**	CH	CH	A-7-6 (40)	A-7-6 (40)	2.42	2.59
**S4**	CL^b^	CL	A-7-6 (10)	A-7-6 (10)	2.44	2.13
**S5**	CH	CH	A-7-5 (260)	A-7-5 (260)	3.00	3.00
**S6**	CL	CL	A-6 (18)	A-6 (18)	2.49	2.38
**S7**	CL	CL	A-6 (12)	A-6 (12)	2.41	2.60

^a^: fat clay,

^b^: lean clay,

^c^: in brackets shown the AASHTO group index

#### Comparison

A qualitative comparison between the LDS and hydrometer results is possible through soil classification systems [4]. As listed in Table 2, both the LDS and hydrometer results designated all the tested samples to the same soil classification in both mainstream systems {USCS (Unified Soil Classification System) and AASHTO (American Association of State Highway and Transportation Officials) including the AASHTO group index}. Therefore, the differences between the two methods, from the geotechnical perspective, are qualitatively insignificant. Moreover, a quantitative comparison was conducted based on the material percentages (silt: 0.075 to 0.005 mm, clay: < 0.002 mm, and colloids: < 0.001 mm). In the case of silt, as shown in Fig 9c, the LDS results are slightly overestimated. This agrees with similar findings reported in the literature [10, 11]. However, the LDS and hydrometer silt results are highly correlated with each other, with a mean (SE) of 7.01%. For clay and colloids, the LDS and hydrometer results are highly correlated with each other, and the mean (SE) was found to decrease as the size decreases (5.49% for colloids, 6.81% for clay), as shown in Fig 9a and 9b, respectively.

## 4. Conclusions

This investigation was aimed to validate the usage of the LDS method as a reliable replacement of the hydrometer method for grain size analysis of fine-grained geomaterials. The findings of this study indicated that the LDS method could be a standalone technique for particle size analysis of fine geomaterials and can reproduce the hydrometer results with appreciable precision. Using the Microtrac S3500 LDS instrument with samples prepared at a concentration of 1.4 g/l in a distilled water solution, a default refractive index value of 1.75, and without the addition of a dispersing agent or sonication, the results were proven to be satisfactory. Based on the liquid limit of the samples, a mathematical model was provided to select the distribution method (either volume or number) to yield the closest results in comparison to the hydrometer method. Based on the provided equation; any sample with LL >= 180% will result in the number method. However, as a recommendation; this intermediate range to be verified by future studies. Nevertheless, the provided equation is valid for the liquid limit range used in the current study.

Further, the sieve analysis for geomaterials is not complicated or time-consuming such as the Hydrometer. Therefore, and as an unsubstantiated opinion; the authors propose a combined sieve-LDS method with a correction of the passing percentage (i.e., the LDS method to be used only for the particles passing the ASTM sieve #200). The differences between the LDS and hydrometer are qualitatively insignificant based on the soil classification criteria (both methods yielded the same soil classification in USCS and AASHTO systems). Quantitatively, the LDS and hydrometer results regarding silt, clay and colloidal contents and fractal dimension are comparable to each other and highly correlated. The LDS method might be considered as a reasonable alternative to the Hydrometer with comparable results that are fast to obtain, continuous (especially for very fine particles), independent of particle density and specific gravity, and generated from the least possible quantity/concentration and efforts. These findings are applicable to the geomaterials range considered in this study, and further evaluation is required to generalize the findings for geomaterials with different properties.

### 4.1. Recommendations for future works

The effect of unbalanced surface charges, suction, mixed origin/size samples, and other physiochemical properties on the scattering results are recommended to be investigated, mainly for clayey materials. The effect of wider range of liquid limit on the selection of the distribution method is also to be investigated.
